# Sudden sensory neural hearing loss is not predictive of myocardial infarction: A longitudinal follow-up study using a national sample cohort

**DOI:** 10.1038/s41598-018-19404-z

**Published:** 2018-01-17

**Authors:** So Young Kim, Songyong Sim, Hyung-Jong Kim, Hyo Geun Choi

**Affiliations:** 1 Department of Otorhinolaryngology-Head & Neck Surgery, CHA Bundang Medical Center, CHA University, Seongnam, Korea; 2 Department of Statistics, Hallym University, Chuncheon, Korea; 3 Department of Otorhinolaryngology-Head & Neck Surgery, Hallym University College of Medicine, Anyang, Korea

## Abstract

The aim of this study was to evaluate the risk of myocardial infarction (MI) in SSNHL subjects with differently matched control groups. The Korean Health Insurance Review and Assessment Service - National Sample Cohort recruited subjects from 2002 to 2013. We used two study designs. In study I, we matched 4,467 SSNHL participants with a control group (17,868 subjects with no history of SSNHL) based on demographic factors (age, sex, income, and region of residence) and medical history (diabetes, dyslipidemia, and hypertension). In study II, we matched 4,467 SSNHL participants with a control group based on only demographic factors. The crude (simple) and adjusted hazard ratios (HRs) of SSNHL with MI were analyzed using the Cox-proportional hazard model. In study I, SSNHL was not associated with increased risk of MI. However, in study II, SSNHL was associated with increased risk of MI (adjusted HR = 1.39 95% CI = 1.00–1.93, P = 0.048). The SSNHL group did not exhibit increased risk of MI when compared to the control group matched by both demographic factors and medical history. However, compared to the control group not matched by medical history, the relative risk of MI was increased in the SSNHL group.

## Introduction

Coronary heart disease, including myocardial infarction (MI), is one of the major causes of mortality worldwide^1^. In the United States, coronary heart disease alone causes approximately 14.3% of all-cause death^2^. Additionally, approximately 160,000 silent MIs occur each year^2^. Moreover, MI patients are susceptible to other comorbidities. Impaired vascular circulation in MI patients may lead to stroke and other end-organ diseases. Target organs with high metabolic rates or scattered vasculature might be especially vulnerable to ischemic injuries. Thus, early detection and prevention of MI is crucial.

The cochlea is a target organ that is susceptible to compromised blood circulation because of high metabolic demands and end-arterial blood supply. Although the pathogenesis of sudden sensorineural hearing loss (SSNHL) is still controversial, ischemia in the inner ear due to posterior circulation, including the vertebrobasilar artery, has been reported to cause SSNHL^3^. In addition to problems in arterial circulation that directly supplies the organ, systemic ischemia, such as that caused by atherosclerosis and hypertension, has been evaluated for an association with SSNHL^4,5^. Several previous studies have demonstrated the risk of MI in SSNHL subjects^6,7^. A population-based case-control study reported an odds ratio of 1.55 for a history of SSNHL in acute myocardial infarction (AMI) subjects (95% confidence interval [95% CI] = 1.30–1.70, P < 0.001)^7^. Based on age groups, 50–64- and ≥65-year-old groups with SSNHL demonstrated adjusted hazard ratios (HRs) of 1.62 and 1.28 for MI, respectively (P = 0.0064 and P < 0.001, respectively)^6^.

Many factors have been reported to be associated with MI and should be considered when investigating the risk of MI^1,8^. Although previous studies have adjusted for several medical histories, including diabetes, dyslipidemia, and hypertension, the SSNHL and control groups were not matched for these medical histories. Thus, the SSNHL groups have shown higher rates of medical histories such as hypertension, diabetes, and dyslipidemia as well as MI compared to the control group^6,7^, which might have impacted the results of the association between SSNHL and MI.

The present study hypothesized that, although SSNHL and MI have a common pathophysiological mechanism of vascular ischemia, the association between SSNHL and MI might be overestimated in prior studies. The other comorbidities of SSNHL subjects might be confounding factors that contribute to the occurrence of MI. Therefore, we designed two different studies to test our hypothesis. In study I, we minimized the confounding effects of other diseases by ensuring that the medical histories of diabetes, dyslipidemia, and hypertension were exactly matched between the SSNHL and control groups. In study II, the medical histories were not matched when selecting the control group, which mimicked the selection process of prior studies. Instead, medical history was statistically adjusted as confounders. Few studies have matched control groups for both medical histories and demographic factors. We used national cohort data, which have been validated in a previous study^9^. In this verified cohort, the diagnosis of MI was reviewed after the diagnosis of SSNHL to delineate the causalities between SSNHL and MI.

## Results

### Study I

The age, sex, income, region of residence, and medical histories of hypertension, diabetes, and dyslipidemia were exactly matched between the SSNHL and control groups in study I (Table 1). The prevalence of MI was 213.2 and 193.3 per 100,000 person-years in the SSNHL and control groups, respectively (P = 0.534). SSNHL was not associated with increased risk of MI in study I (adjusted HR = 1.10, 95% CI = 0.81–1.50, P = 0.535) (Table 2). In both the <50- and ≥50-year-old subgroups, SSNHL was not associated with increased risk of MI (Table 3). In another subgroup analysis, the duration of the follow-up did not influence the risk of MI in the SSNHL groups.

**Table 1:** General Characteristics of Participants in Study I and Study II (matched 1:4).

Characteristics	Study I			Study II		
	SSNHL (n, %)	Control group (n, %)	P-value	SSNHL (n, %)	Control group (n, %)	P-value
Age (years old)			1.000			1.000
5–9	2 (0.0)	8 (0.0)		2 (0.0)	8 (0.0)	
10–14	22 (0.5)	88 (0.5)		22 (0.5)	88 (0.5)	
15–19	79 (1.8)	316 (1.8)		79 (1.8)	316 (1.8)	
20–24	109 (2.4)	436 (2.4)		109 (2.4)	436 (2.4)	
25–29	140 (3.1)	560 (3.1)		140 (3.1)	560 (3.1)	
30–34	229 (5.1)	916 (5.1)		229 (5.1)	916 (5.1)	
35–39	271 (6.1)	1,084 (6.1)		271 (6.1)	1,084 (6.1)	
40–44	381 (8.5)	1,524 (8.5)		381 (8.5)	1,524 (8.5)	
45–49	389 (8.7)	1,556 (8.7)		389 (8.7)	1,556 (8.7)	
50–54	511 (11.4)	2,044 (11.4)		511 (11.4)	2,044 (11.4)	
55–59	542 (12.1)	2,168 (12.1)		542 (12.1)	2,168 (12.1)	
60–64	482 (10.8)	1,928 (10.8)		482 (10.8)	1,928 (10.8)	
65–69	395 (8.8)	1,580 (8.8)		395 (8.8)	1,580 (8.8)	
70–74	414 (9.3)	1,656 (9.3)		414 (9.3)	1,656 (9.3)	
75–79	294 (6.6)	1,176 (6.6)		294 (6.6)	1,176 (6.6)	
80–84	133 (3.0)	532 (3.0)		133 (3.0)	532 (3.0)	
85+	74 (1.7)	296 (1.7)		74 (1.7)	296 (1.7)	
Sex			1.000			1.000
Male	1,967 (44.0)	7,868 (44.0)		1,967 (44.0)	7,868 (44.0)	
Female	2,500 (56.0)	10,000 (56.0)		2,500 (56.0)	10,000 (56.0)	
Income		1.000			1.000	
1 (lowest)	79 (1.8)	316 (1.8)		79 (1.8)	316 (1.8)	
2	282 (6.3)	1128 (6.3)		282 (6.3)	1128 (6.3)	
3	296 (6.6)	1,184 (6.6)		296 (6.6)	1,184 (6.6)	
4	262 (5.9)	1,048 (5.9)		262 (5.9)	1,048 (5.9)	
5	309 (6.9)	1,236 (6.9)		309 (6.9)	1,236 (6.9)	
6	325 (7.3)	1,300 (7.3)		325 (7.3)	1,300 (7.3)	
7	431 (9.6)	1,724 (9.6)		431 (9.6)	1,724 (9.6)	
8	423 (9.5)	1,692 (9.5)		423 (9.5)	1,692 (9.5)	
9	571 (12.8)	2,284 (12.8)		571 (12.8)	2,284 (12.8)	
10	700 (15.7)	2,800 (15.7)		700 (15.7)	2,800 (15.7)	
11 (highest)	789 (17.7)	3,156 (17.7)		789 (17.7)	3,156 (17.7)	
Region of residence			1.000			1.000
Urban	2,057 (46.0)	8,228 (46.0)		2,057 (46.0)	8,228 (46.0)	
Rural	2,410 (54.0)	9,640 (54.0)		2,410 (54.0)	9,640 (54.0)	
Hypertension			1.000			<0.001*
Yes	1,674 (37.5)	6,696 (37.5)		1,674 (37.5)	5,837 (32.7)	
No	2,793 (62.5)	11,172 (62.5)		2,793 (62.5)	12,031 (67.3)	
Diabetes			1.000			<0.001*
Yes	970 (21.7)	3,880 (21.7)		970 (21.7)	2,930 (16.4)	
No	3,497 (78.3)	13,988 (78.3)		3,497 (78.3)	14,938 (83.6)	
Dyslipidemia			1.000			<0.001*
Yes	1,403 (31.4)	5,612 (31.4)		1,403 (31.4)	4,312 (24.1)	
No	3,064 (68.6)	12,256 (68.6)		3,064 (68.6)	13,556 (75.9)	
MI			0.534			0.002*
Yes	51 (1.1)	185 (1.0)		51 (1.1)	124 (0.7)	
No	4,416 (98.9)	17,683 (99.0)		4,416 (98.9)	17,744 (99.3)	

SSNHL: sudden sensory neural hearing loss.

MI: myocardial infarction.

*Chi-square test or Fisher’s exact test. Significance at P < 0.05.

**Table 2:** Crude and adjusted hazard ratios (95% confidence interval) of sudden sensory neural hearing loss (SSNHL) for myocardial infarction in Study I and Study II.

SSNHL	Hazard ratios			
	Crude	P-value	Adjusted^†^	P-value
Study I				
SSNHL	1.10 (0.81–1.50)	0.536	1.10 (0.81–1.50)	0.535
Control	1.00		1.00	
Study II				
SSNHL	1.66 (1.19–2.29)	0.002^*^	1.39 (1.00–1.93)	0.048^*^
Control	1.00		1.00	

^*^Cox-proportional hazard regression model, Significance at P < 0.05.

^†^Adjusted model for age, sex, income, region of residence, hypertension, diabetes, and dyslipidemia histories.

**Table 3:** Crude and adjusted hazard ratios (95% confidence interval) of sudden sensory neural hearing loss (SSNHL) for myocardial infarction according to age group and follow up periods in Study I.

SSNHL	Hazard ratios			
	Crude	P-value	Adjusted^†^	P-value
Age <50 years old (n = 8,110)				
SSNHL	1.23 (0.40–3.78)	0.716	1.21 (0.40–3.72)	0.737
Control	1.00		1.00	
Age ≥50 years old (n = 14,225)				
SSNHL	1.09 (0.79–1.51)	0.590	1.09 (0.80–1.51)	0.587
Control	1.00		1.00	
Follow up ≥3 years (n = 16,615)				
SSNHL	1.13 (0.81–1.57)	0.467	1.13 (0.81–1.57)	0.468
Control	1.00		1.00	
Follow up ≥5 years (n = 12,185)				
SSNHL	1.12 (0.80–1.59)	0.509	1.12 (0.79–1.59)	0.511
Control	1.00		1.00	

^*^Cox-proportional hazard regression model, Significance at P < 0.05.

^†^Adjusted model for age, sex, income, region of residence, hypertension, diabetes, and dyslipidemia histories.

### Study II

The age, sex, income, and region of residence were exactly matched between the SSNHL and control groups in study II (Table 1). However, the rates of a medical history of hypertension, diabetes, and dyslipidemia were higher in the SSNHL group than the control group. The prevalence of MI was 213.2 and 132.3 per 100,000 person-years in the SSNHL and control groups, respectively (P = 0.002). In study II, SSNHL was associated with increased risk of MI (adjusted HR = 1.39, 95% CI = 1.00–1.93, P = 0.048) (Table 2). Both the <50- and ≥50-year-old groups demonstrated a high risk of MI (crude HR = 5.23, 95% CI = 1.20–23.87, P = 0.028 for the <50-year-old group; crude HR = 1.56, 95% CI = 1.11–2.18, P = 0.013 for the ≥50-year-old group) (Table 4). The <50-year-old group with SSNHL exhibited an increased risk of MI (adjusted HR = 4.92 95% CI = 1.08–22.29, P = 0.039). Another subgroup analysis based on follow-up duration showed that SSNHL was associated with increased risk of MI (crude HR = 1.57, 95% CI = 1.12–2.21, P = 0.010 for the ≥3-year follow-up group; crude HR = 1.60, 95% CI = 1.12–2.30, P = 0.014 for the ≥5-year follow-up group). However, in the model adjusted for age, sex, income, region of residence, and medical history, SSNHL was not associated with increased risk of MI in both subgroups based on follow-up duration.

**Table 4:** Crude and adjusted hazard ratios (95% confidence interval) of sudden sensory neural hearing loss (SSNHL) for myocardial infarction according to age group and follow up periods in Study II.

SSNHL	Hazard ratios			
	Crude	P-value	Adjusted^†^	P-value
Age <50 years old (n = 8,110)				
SSNHL	5.23 (1.20–23.87)	0.028^*^	4.92 (1.08–22.29)	0.039^*^
Control	1.00		1.00	
Age ≥50 years old (n = 14,225)				
SSNHL	1.56 (1.11–2.18)	0.013^*^	1.32 (0.94-0.185)	0.111
Control	1.00		1.00	
Follow up ≥3 years (n = 16,615)				
SSNHL	1.57 (1.12–2.21)	0.010^*^	1.32 (0.94–1.86)	0.114
Control	1.00		1.00	
Follow up ≥5 years (n = 12,185)				
SSNHL	1.60 (1.12–2.30)	0.014^*^	1.35 (0.94–1.95)	0.105
Control	1.00		1.00	

^*^Cox-proportional hazard regression model, Significance at P < 0.05.

^†^Adjusted model for age, sex, income, region of residence, hypertension, diabetes, and dyslipidemia histories.

## Discussion

In study I, SSNHL was not associated with increased risk of MI in the present study when the control group was matched for age, sex, income, region of residence, and medical histories of hypertension, diabetes, and dyslipidemia. However, when the control group was matched for only age, sex, income, and region of residence but not for medical history (study II), the SSNHL group was associated with increased risk of MI, even in the adjusted statistical models for medical history. The present study improved upon the previous findings on the risk of MI among patients with SSNHL by demonstrating different findings of MI risk depending on the matching processes for the sampling of the control groups.

Previous studies on the relationship between hearing loss and cardiovascular diseases have demonstrated contradictory results. An increased risk of cardiovascular disease in SSNHL subjects has been reported in several previous studies. Due to the lack of collateral blood supply and the high metabolic demands of cochlear hair cells, the cochlea is suggested to be susceptible to hypoxia or ischemic vascular diseases, such as hypertension, diabetes, dyslipidemia, and MI^10^. Moreover, several previous studies have suggested that several vascular factors related to coagulation, the nervous system, and endothelial function are impaired in SSNHL patients^11–13^. On the other hand, other previous studies have reported no evident association between hearing loss and cardiovascular disease^14^. A prospective study reported that the recovery of hearing threshold in SSNHL subjects was not affected by cardiovascular risk factors^15^. Variations in study population, the type of cardiovascular disease, and the etiologies of hearing loss, such as noise-induced, age-related, and microvascular disease-related hearing loss, as well as the analytical methods could cause these discordances among studies.

In this study, we did not observe an association of SSNHL with increased risk of MI. This finding could be explained by differences in the control groups. The prevalence of MI in the SSNHL group was 213.2 per 100,000 person-years, which was comparable to the prior report of 192.7 per 100,000 person-years^6^. However, there were differences in the control groups regarding medical history. The control group in study I demonstrated the same rate of hypertension, diabetes, and dyslipidemia as the SSNHL group as they were matched for these factors. On the other hand, the control group in study II showed a relatively lower rate of hypertension, diabetes, and dyslipidemia compared to the SSNHL group. Like study II, previous studies have also demonstrated higher rates of certain medical histories in the SSNHL group than in the control group^6^. Although medical histories were statistically adjusted in those previous studies, the study groups were not matched for medical history. Unmatched medical histories could result in higher rates of hypertension, diabetes, and dyslipidemia in the SSNHL group, thereby resulting in selection bias. Because hypertension, diabetes, and dyslipidemia are related to MI, these medical histories could confound the relationship between MI and SSNHL in these prior studies^6,7^ despite statistical adjustments.

In addition, it might be possible that SSNHL is only partially linked with cardiovascular diseases. There are many causes or factors related to SSNHL. Approximately 71.0% of SSNHL are idiopathic cases, followed by infectious (12.8%), otologic (4.7%), traumatic (4.2%), and vascular or hematologic (2.8%) causes^16^. For instance, degenerative changes in the inner ear, in addition to ischemic changes in the strial vascularis, might contribute to hearing loss. Although low-frequency hearing loss was proposed to be related to cardiovascular risk factors, most studies reporting this were conducted in an elderly population with presbycusis^14,17,18^. The sparse distribution of strial vascularis in cochlear apex lesions are thought to be vulnerable to ischemia. However, degenerative changes cannot be excluded in these studies on elderly populations. In animal models, degenerative and atrophic changes in the strial vascularis induced hearing loss^19^.

There is several strengths in the present study. The SSNHL was diagnosed using multiple criteria. In addition to ICD-10 codes, the results of pure tone audiometry and treatment with steroids were investigated in this study. As a result, our annual incidence of SSNHL was approximately 42.6/100,000 persons (5,244/1,025,340 persons for 12 years), which was comparable with previous studies reporting an incidence of 4–160/100,000 persons^20^. For the diagnosis of MI, the ICD-10 code I21 was used as described in a previous study^21^. However, the incidence of MI (132.3–213.2/100,000 person-years) was higher in this study than that in a previous study (29.4–41.6/100,000 person-years) because that study evaluated hospitalized MI patients^21^. Consistent with our data, another study previously reported an annual MI incidence of 118.4/100,000 persons using multiple ICD-10 codes (I21, I22, I23, I259, and I251)^22^. The current study followed up for a diagnosis of MI after the diagnosis of SSNHL. Thus, a temporal relationship between SSNHL and MI could be elucidated. Moreover, repeat analyses with different control groups helped uncover the confounding effects of medical history on the association between SSNHL and MI. The cohort population of 1,025,340 allowed this study to retrieve two control groups, matching for medical history and/or demographics. The control group matched for medical history provided different results compared with previous studies in which the control groups were not matched for medical history. Moreover, to prevent selection bias in the control group, randomization was conducted before selecting the control subjects.

However, the present results should be interpreted with some limitations. Although the present study used control groups matched for several medical histories in addition to demographic factors, there are still possible confounders that were not considered, including cerebrovascular diseases and thrombotic risk factors. In addition, the audiometric patterns of SSNHL could not be delineated in data of a large population cohort. The degree or severity of MI also could not be assessed in this study.

## Conclusion

SSNHL was not associated with increased risk of MI when the control group was matched for age, sex, income, region of residence, and medical histories of diabetes, dyslipidemia, and hypertension.

## Materials and Methods

### Study Population and Data Collection

The Ethics Committee of Hallym University (2014-I148) approved the use of these data. All methods were carried out in accordance with guidelines and regulations of the Ethics Committee of Hallym University. Written informed consent was exempted by the institutional review board.

This national cohort study relied on data from the Korean Health Insurance Review and Assessment Service - National Sample Cohort (HIRA-NSC). The Korean National Health Insurance Service (NHIS) selects samples directly from the entire population database to prevent non-sampling errors. Approximately 2% of the samples (one million) were selected from the entire Korean population (50 million). These selected data can be classified at 1,476 levels (age [18 categories], sex [2 categories], and income level [41 categories]), and randomized stratified systematic sampling methods via proportional allocation are used to represent the entire population. After data selection, the appropriateness of the sample was verified by a previous study^9^. The details of the methods used to perform these procedures are provided by the National Health Insurance Sharing Service^23^. This cohort database included (i) personal information, (ii) health insurance claim codes (procedures and prescriptions), (iii) diagnostic codes using the International Classification of Disease-10 (ICD-10), (iv) death records from the Korean National Statistical Office (using the Korean Standard Classification of Disease), (v) socio-economic data (residence and income), and (vi) medical examination data for each participant from 2002 to 2013.

Because all Korean citizens are recognized by a 13-digit resident registration number from birth to death, exact population statistics can be determined using this database. It is mandatory for all Koreans to enroll in the NHIS. All Korean hospitals and clinics use the 13-digit resident registration number to register individual patients in the medical insurance system. Therefore, the risk of overlapping medical records is minimal, even if a patient moves from one place to another. Moreover, all medical treatments in Korea can be tracked without exception using the HIRA system. In Korea, notice of death to an administrative entity is legally required before a funeral can be held. Causes of death and the date are recorded by medical doctors on a death certificate.

### Participant Selection

Out of 1,025,340 cases with 114,369,638 medical claim codes, we included participants who were diagnosed with SSNHL, ICD-10: H912). Among them, we included only the participants who underwent an audiometry exam (claim code: E6931-E6937, F6341-F6348) and were treated with steroids. From 2002 to 2012, 4,571 SSNHL participants were selected, and the participants were followed up for at least one year and up to 12 years.

Following the methods described by a previous study^21^, patients with a history of MI were identified using ICD-10 codes (I21). MI participants were defined as those who were treated ≥1 time (n = 11,638) for MI-related issues between 2002 and 2013.

#### Study I

The SSNHL participants were matched 1:4 with control participants who were never diagnosed with SSNHL from 2002 to 2013. The control groups were selected from the mother population (n = 1,025,340). The matches were processed for age, group, sex, income group, region of residence, and medical history (hypertension, diabetes, and dyslipidemia). To prevent selection bias when selecting the matched participants, the control group participants were sorted using a random number order, and they were then selected from top to bottom. It was assumed that the matched control participants were evaluated at the same time as each matched SSNHL participant (index date). Therefore, the control participants who died before the index date were excluded. In both the SSNHL and control groups, participants who had a history of MI before the index date were also excluded. In the SSNHL group, 53 participants were excluded. The SSNHL participants for whom we could not identify enough matched control participants were excluded (n = 51). Finally, 1:4 matching resulted in the inclusion of 4,467 SSNHL participants and 17,868 control participants.

#### Study II

**Figure 1:**
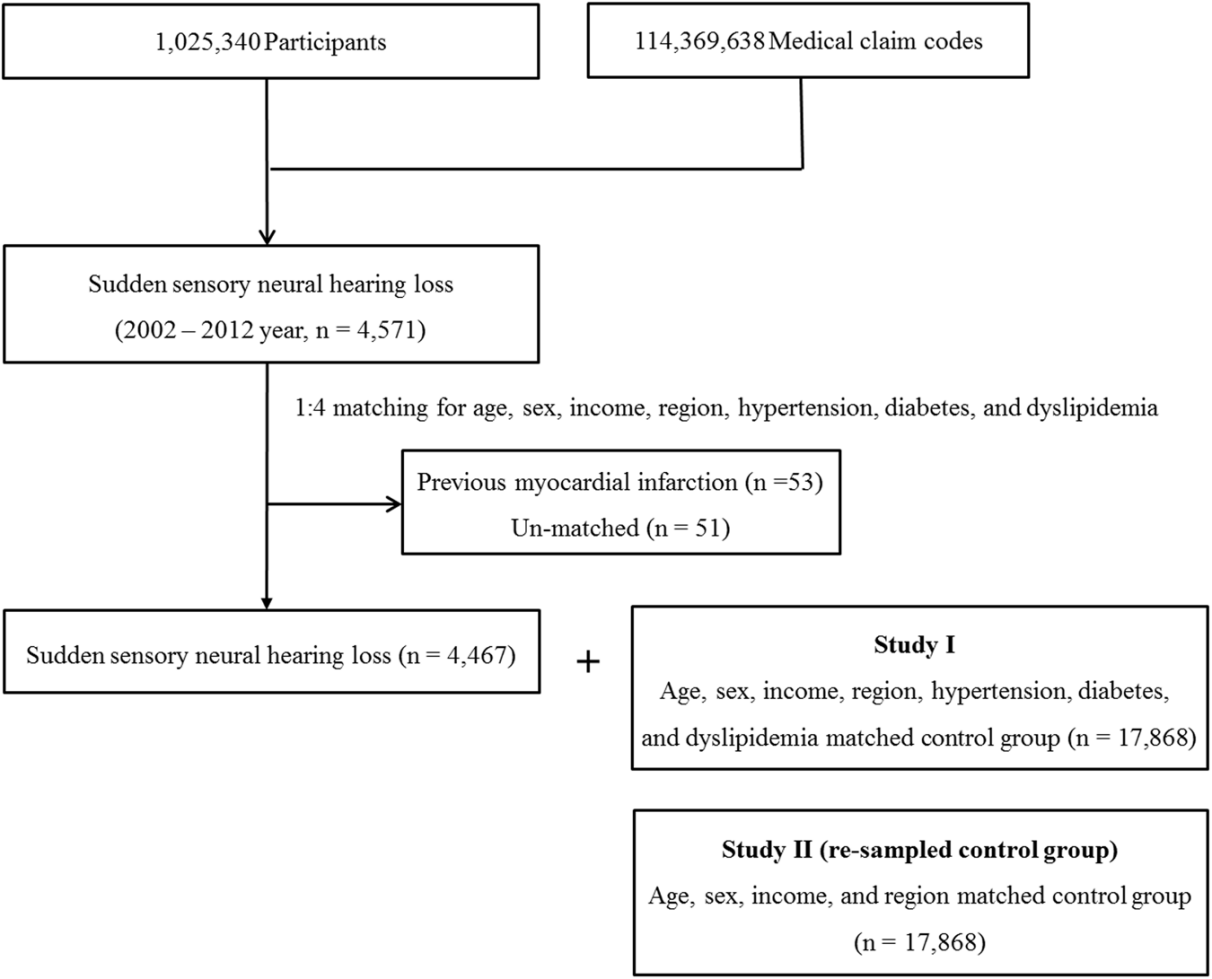
A schematic illustration of the participant selection process that was used in the present study. Out of a total of 1,025,340 participants, 4,467 SSNHL participants were matched with control participants for age, group, sex, income group, region of residence, and medical history (study I) and were rematched with a new group of control participants for age, group, sex, income group, and region of residence (study II).

In this study, the 4,467 SSNHL participants identified in the previous study were rematched with control participants from the mother population (n = 1,025,340). The control group was again arranged in random order, and they were then selected from top to bottom. This time, the control participants were matched for age, group, sex, income group, and region of residence but not for medical history (hypertension, diabetes, and dyslipidemia). Finally, 1:4 matching resulted in the inclusion of 4,467 SSNHL participants and 17,868 control participants (Fig. 1).

### Variables

The age groups were classified using 5-year intervals: 0–4, 5–9, 10–14…, and 85+ years old. A total of 18 age groups were designated. Because of the limited participants, only 17 age groups were defined (no 0–4 age group). The income groups were initially divided into 41 classes (one health aid class, 20 self-employment health insurance classes, and 20 employment health insurance classes). These groups were recategorized into 11 classes (class 1 [lowest income]−11 [highest income]). The region of residence was divided into 16 areas based on the administrative district. These regions were regrouped into urban (Seoul, Busan, Daegu, Incheon, Gwangju, Daejeon, and Ulsan) and rural (Gyeonggi, Gangwon, Chungcheongbuk, Chungcheongnam, Jeollabuk, Jeollanam, Gyeongsangbuk, Gyeongsangnam, and Jeju) areas.

The medical histories of participants were evaluated using ICD-10 codes. For the accuracy of diagnosis, hypertension (I10 and I15), diabetes (E10-E14), and dyslipidemia (E78) were confirmed if the participants were treated ≥2 times.

### Statistical Analyses

Chi-square test or Fisher’s exact test was used to compare the general characteristics between the SSNHL and control groups.

To analyze the HRs of SSNHL with MI in each study (I and II), the Cox-proportional hazard model was used. In this analysis, the crude (simple) and adjusted (age, sex, income, region of residence, hypertension, diabetes, and dyslipidemia) models were used. HRs and 95% CIs were calculated.

For the subgroup analysis, we stratified the participants by age (<50 years old vs. ≥50 years old). Because some participants were followed up for only one year (SSNHL participants in 2012), we included only the ≥3-year and ≥5-year follow-up groups.

Two-tailed analyses were conducted. The results were statistically analyzed using SPSS v. 21.0 (IBM, Armonk, NY, USA).
